# Less Is More?—A Feasibility Study of Fluid Strategy in Critically Ill Children With Acute Respiratory Tract Infection

**DOI:** 10.3389/fped.2019.00496

**Published:** 2019-12-10

**Authors:** Sarah A. Ingelse, Vincent G. Geukers, Monique E. Dijsselhof, Joris Lemson, Reinout A. Bem, Job B. van Woensel

**Affiliations:** ^1^Department of Pediatric Intensive Care, Emma Children's Hospital, Amsterdam UMC, University of Amsterdam, Amsterdam, Netherlands; ^2^Department of Dietetics, Emma Children's Hospital, Amsterdam UMC, Amsterdam, Netherlands; ^3^Department of Intensive Care, Radboud University Medical Center, Nijmegen, Netherlands

**Keywords:** fluid therapy, critical care, respiratory tract infection, bronchiolitis, child health, feasibility studies

## Abstract

**Background:** Fluid overload is common in critically ill children and is associated with adverse outcome. Therefore, restricting fluid intake may be beneficial. This study aims to study the feasibility of a randomized controlled trial (RCT) comparing a conservative to a standard, more liberal, strategy of fluid management in mechanically ventilated pediatric patients with acute respiratory tract infection (ARTI).

**Methods:** This is a feasibility study in a single, tertiary referral pediatric intensive care unit (PICU). Twenty-three children receiving mechanical ventilation for ARTI, without ongoing hemodynamic support, admitted to the PICU of the Emma Children's Hospital/Amsterdam UMC between 2016 and 2018 were included. Patients were randomized to a conservative (<70% of normal intake) or standard (>85% of normal intake) fluid strategy, which was kept throughout the period of mechanical ventilation.

**Results:** Primary endpoints were adherence to fluid strategy and safety parameters such as calorie and protein intake. Secondary outcomes were cumulative fluid intake (CFI) and cumulative fluid balance (CFB) on day 3. In the conservative group, in 75% of the mechanical ventilation days patients achieved their target fluid intake. Median [25th−75th percentiles] calorie intake over all mechanical ventilation days was 67.9 [51.5–74.0] kcal/kg/day in the conservative vs. 67.2 [58.0–75.2] kcal/kg/day in the standard group (*p* = 0.878). Protein intake was 1.6 [1.3–1.8] gr protein/kg in the conservative and 1.5 [1.2–1.7] gr protein/kg in the standard group (*p* = 0.598). No adverse effects on hemodynamics or electrolyte imbalances were noted. Mean (±SD) CFI on day 3 was 262.3 (±58.9) ml/kg in the conservative group vs. 360.5 (±52.6) ml/kg in the standard fluid group (*p* < 0.001), which did not result in a lower CFB.

**Conclusions:** A conservative fluid strategy in mechanically ventilated children with ARTI seems feasible, without limiting metabolic needs. However, in our study a conservative fluid strategy surprisingly did not reduce the degree of fluid overload. This study aids the design and sample size calculation of a future larger multicenter RCT, in which we need to redefine the target fluid strategy, possibly by even further fluid restriction and early initiation of active diuresis.

**Clinical Trial Registration**: ClinicalTrials.gov, identifier: NCT02989051.

## Background

Fluid overload is a major problem in critically ill patients and is gaining increasing attention in both research and clinical practice. Importantly, body fluid mostly accumulates early in the disease process (1, 2), which is believed to occur due to multiple factors, such as overzealous intravenous fluid loading, pro-inflammatory injury with systemic capillary leak and cardiopulmonary dysfunction during critical illness (3, 4). Fluid overload results in interstitial fluid retention, including formation of pulmonary edema, compromising alveolar-capillary oxygen diffusion. Numerous studies have shown that—in both adults and children—fluid overload, or extreme positive cumulative fluid balance (CFB), has adverse effects on outcome, such as a longer duration of mechanical ventilation and even higher mortality rates (2, 5–8). Of great interest, a large randomized controlled trial (RCT) in adult acute respiratory distress syndrome patients has shown a conservative fluid management regimen to lead to more ventilator-free days (9, 10).

As evidence in the pediatric population up to this point is exclusively mounted through observational studies, it becomes clear that there is a great need for prospective testing in children (6, 11). However, studies in children present with considerable challenges such as small patient populations and different ethical issues. For example, children cannot give their own consent and are more vulnerable than adults. Moreover, critically ill children are prone to cumulative energy and protein deficits during pediatric intensive care unit (PICU) admission, due to both insufficient amounts of prescribed calories and unreliable predictive requirement equations (12, 13), with 50% of cumulative protein energy malnutrition developing in the first 48 h of admission (12). Negative protein balance with loss of lean body mass is associated with a higher risk of infections, persisting critical illness, and increased length of stay in the PICU (14, 15). Restrictive fluid management may add to this risk. In order to reach positive protein balance, the minimum nutritional requirements for critically ill children are found to be 57 kCal/kg/day and 1.5 g/kg/day protein intake (16). Along with the logistical difficulty of reaching adequate study sample sizes, these issues together raise the need for feasibility studies in critically ill children.

We performed a study to determine the feasibility and safety of a larger multicenter RCT comparing current fluid maintenance protocols with an early conservative fluid management strategy. Primary endpoints were adherence to fluid strategy and safety parameters. We specifically chose a highly prevalent patient cohort of mechanically ventilated critically ill children with acute respiratory tract infection (ARTI), not necessarily characterized by major hemodynamic instability and capillary leak, in order to steer toward a general PICU fluid management protocol.

## Methods

This is a single center feasibility study in patients admitted to the PICU of the Emma Children's Hospital/Amsterdam UMC, The Netherlands. Our PICU is a 12-bed, tertiary unit, serving the greater Amsterdam area in The Netherlands. Patients were enrolled between September 2016 and April 2018 mostly by the primary researcher with the help of the attending physicians in the PICU.

Patients were randomized in a 1:1 allocation ratio to a conservative fluid regimen or a standard, more liberal, fluid regimen in randomly permuted blocks of lengths 4 and 6 with the use of an encrypted and automated website (Sealed Envelope™). A Safety Monitoring board was set up to monitor monthly progress and possible relation with adverse events in both study arms.

### Sample Size

Although this is a feasibility trial and therefore formal sample size calculations are more challenging, we chose to base our sample size calculation on the CFB on day 3 (CFB3), as this one of the primary outcomes for the future larger set-up and a parameter most often used in literature to describe fluid overload. Earlier work by our group showed a mean (±SD) CFB3 of mechanically ventilated patients with acute respiratory failure due to severe bronchiolitis of +97.9 (±49.2) ml/kg. Aiming for a 50% decrease in CFB3 in patients who receive a conservative fluid treatment vs. standard treatment, a sample size of 17 in each group will have 80% power to detect a difference in means of 50% (the difference between a group 1 mean of 100 ml/kg and a group 2 mean of 50 ml/kg) assuming that the common standard deviation is 49.2 using a two group *t*-test with a 0.05 two-sided significance level. In literature, sample size in pilot studies is recommended to be a minimum of 12 participants per treatment arm (17, 18) confirming our calculated sample size to be of sufficient size.

### Inclusion Criteria

Patients below 10 years of age were eligible when they were intubated and mechanically ventilated for a (suspected) ARTI, with an anticipated duration of mechanical ventilation of at least 72 h at enrolment. Enrolment into the study protocol needed to be fulfilled within 12 h after start of mechanical ventilation.

### Exclusion Criteria

Exclusion criteria were use of previous or maintenance diuretic treatment at enrolment, ongoing (fluid) resuscitation at enrolment, acute kidney injury with the need for renal replacement therapy and patients in need of a particular fluid regimen (i.e., for their medical history such as cardiovascular disease and/or congenital heart disease).

### Study Protocol

Age-dependent fluid strategies are defined by international guidelines for healthy children (19, 20). Yet, fluid delivery typically fluctuates and specifically fluid during the maintenance phase of treatment of critically ill patients often far exceeds the normal fluid requirements (21). Based on clinical experience and feasibility, in this study protocol, we aimed for <70% of recommended intake in our intervention group (conservative fluid regimen), compared to at least 85% of recommended intakes in the control group (standard, liberal fluid regimen). In both groups of our study we thus set strict fluid targets for each patient based on their admission weight (Supplementary Table 1). Fluid treatment regimens were set to guarantee the caloric and protein requirements as described by Bechard et al. (16). Study days were defined as each day the patient was still on invasive mechanical ventilation. As soon as patients were extubated, the study protocol did not apply to them anymore. During the study period, the attending physician was free to start diuretic treatment when clinically needed, or even make adaptations in the allocated fluid treatment when there were valid clinical reasons to do so.

### Data Collection

Data were prospectively collected from the patient clinical data management system. Allocation to one of the fluid arms was not blinded for the attending physicians, dietitians or researchers, as we wanted fluid and feeding calculations to be as accurate as possible in both fluid arms and it is difficult to blind for a difference in fluid volume.

#### Primary Outcomes

Adherence was observed for every study day for each patient and median adherence over all study days was calculated in a percentage of the total number of study days. Reasons for non-adherence were examined for the conservative fluid strategy as this is the most challenging intervention with respect to limiting fluid volume for medication and nutrition. Safety parameters were calorie and protein intake, the need for interventions such as fluid boluses or administration of diuretics. Calorie and protein intake were assessed daily with the help of an experienced dietician. To assess hemodynamic stability in either fluid strategy, blood pressure, and heart rate were monitored continuously. In order to evaluate electrolyte imbalances, available sodium, potassium, and chloride assays were checked for too low or high values. An electrolyte imbalance was scored if the levels of the respective electrolytes were too high or low for more than 1 day consecutively during admission (normal ranges used: sodium 135–145 mmol/L; potassium 3.5–5.2 mmol/L; chloride 96–111 mmol/L). To assess the occurrence of acute kidney injury (AKI) the KDIGO AKI criteria were used (22) by using the urine output criteria. Serum creatinine was not used as this was not readily available at baseline in many children.

#### Secondary Outcomes

Secondary outcomes were (cumulative) fluid intake, fluid output and fluid balance, which were collected daily for the whole period of mechanical ventilation. Fluid intake included intravenously administered fluids and medication, parenteral and enteral feeding, oral medication, and blood products. Fluid output included urine output, stools, gastric aspirate, blood loss, fluid loss from drains, and losses from other body cavities. Fluid balance was calculated over every 24 h as intake minus output per kilogram (kg) body weight on admission. The CFB3 was calculated, as most fluid overload occurs in the first 72 h of start of mechanical ventilation and is therefore one of the most used parameters in literature to describe fluid overload (6). In the patient data management system, ventilation and oxygenation variables were recorded continuously and validated at least hourly by the attending nurses caring for that patient. The Pediatric Index of Mortality (PIM) 2 score was calculated from each patient to assess for severity of illness. To assess the severity of oxygenation defect in our patients, the oxygen saturation index (OSI) was calculated each morning as OSI = ([Paw x FiO_2_]/SpO_2_) × 100, only when SpO_2_ was below 97% (23).

#### Adaptation to Trial Protocol

The primary and secondary outcomes were slightly altered from earlier trial protocol, because the most important outcomes are adherence and safety parameters for this feasibility study. However, for a future, larger RCT, fluid intake and balance will be of more significance. As this is a feasibility study with small numbers, these fluid intake and balance cannot be studied as (primary) outcome measures.

### Statistical Analysis

All analyses were performed according to the intention-to-treat principle. As this was a feasibility study, all analyses listed are exploratory and used to inform future trial designs in this area. A two-sided *p*-value of 0.05 was considered as statistically significant. Statistical analyses were performed using SPSS Statistics (V.25) and R statistical programming software (24) and the “lme4” (25) package was used for mixed-effects model analyses. A linear mixed-effects model was used to analyse the effect of time (as a continuous variable) and fluid strategy on blood pressure, heart rate, and oxygen saturation index. The model uses a group variable being the intervention or control group, with a group:time interaction, and a random intercept and slope per subject. As feasibility parameters, we compared adherence and calorie- and protein intake between groups. Safety parameters compared between groups are cumulative furosemide dose, cumulative diuresis, intervention of fluid boluses given, occurrence of AKI, blood pressure, heart rate and electrolyte imbalances. Blood pressure and heart rate were, as well as being assessed over time in the linear mixed model, also each assessed as a summary measure and compared between groups. Summary measures were calculated as the difference in blood pressure and heart rate between day 3 and 1 was calculated and this delta was assessed between groups. Secondary outcomes compared between groups are fluid intake and balance and if available, increase in body weight. Moreover, duration of mechanical ventilation was compared between groups. Patient characteristics are described by descriptive statistics. Student's *t*-test or Mann-Whitney *U* tests are used to compare continuous variables based on whether data was normally distributed or not. Cohen's *d* was used for calculating effect size for parametric data by calculating the mean difference between the two groups and then dividing the result by the pooled standard deviation (SD). Confidence interval of the difference for non-parametric analyses were calculated using *CIA* (Confidence Interval Analysis 2.2.0) software. Fisher's exact test was used for categorical variables and proportions, as expected counts were lower than 5. Data are expressed as means (±SD), medians [25–75th percentiles] or proportions as appropriate.

## Results

### Patient Characteristics

**Figure 1:**
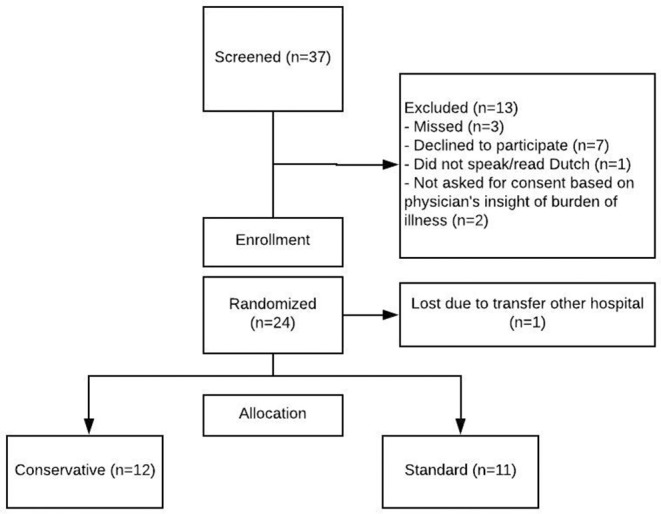
Enrolment and randomization. Patients were screened if at least one of the inclusion criteria (respiratory insufficiency due to possible viral infection) was met. This figure shows reasons for exclusion and subsequent enrolment for patients who consented to participation.

During the study period 37 patients were screened for enrollment. In Figure 1, the screening and randomization process is presented. Twenty four patients gave their consent for inclusion, but one patient did not start the study protocol due to referral to another hospital. Therefore, 23 patients were included and enrolled in the study protocol. Of these, 12 were randomized to the conservative fluid arm and 11 in the standard fluid arm. Table 1 shows their baseline and clinical characteristics.

**Table 1:** Baseline demographic and clinical characteristics of mechanically ventilated patients with acute respiratory tract infection.

**Variable**	**Overall (*N* = 23)**	**Fluid strategies**	
		**Conservative group (*N* = 12)**	**Standard group (*N* = 11)**
Age (months), median [25–75th percentiles]	2.7 [0.8–10.1]	3.4 [0.8–11.5]	2.7 [0.8–6.7]
Sex, male, *N* (%)	13 (57)	6 (50)	7 (64)
Admission weight in kg, median [25–75th percentiles]	5.5 [3.9–8.2]	5.7 [4.3–9.8]	5.5 [3.4–8.2]
History of chronic illness^a^, N (%)	4 (17)	4 (33)	0 (0)
RSV positive, *N* (%)	17 (74)	11 (92)	6 (55)
Bacterial (super)infection^b^, *N* (%)	9 (39)	6 (50)	3 (27)
Pediatric Index of Mortality 2 score, median [25–75th percentiles]	1.3 [1.1–2.1]	1.3 [1.0–2.2]	1.5 [1.1–2.1]
Oxygen saturation index at day of admission, median [25–75th percentiles]	6.1 [4.0–7.4]	6.6 [3.9–8.1]	6.1 [4.1–6.5]

a *Chronic illness included prematurity with bronchopulmonary dysplasia, muscle-eye-brain disease, and epilepsy*.

b *Positive cultures of tracheal aspirate or bronchial lavage fluid included one or more of the following bacteria: Haemophilus influenzae, Moraxella catarrhalis, Streptococcus pneumoniae, Staphylococcus aureus*.

Median age was 2.7 months [25–75^th^ percentiles 0.8–10.1] and was distributed evenly among the treatment groups. There was no meaningful difference in the sex distribution between fluid strategies. Patients had a median [25–75th percentiles] admission weight of 5.5 kg [3.9–8.2], which was also similar in both fluid strategy groups. There were no other important differences of disease severity parameters, such as the PIM2 score or OSI, between the fluid strategy groups (Table 1).

### Feasibility

In this feasibility study, an enrollment rate of 62% was reached (23/37 patients screened for participation). The main reasons for non-inclusion were because parents declined participation. Three patients were missed, and two patients were not asked for permission because the attending physician considered the emotional burden of their child's disease already too demanding to ask for consent. During 75% of the study days, mechanically ventilated children in the conservative group received fluid according to their target intake. There was no significant difference in adherence between both fluid management groups (Table 2).

**Table 2:** Feasibility and safety parameters per fluid strategy.

**Parameter**	**Conservative group (*n* = 12)**	**Standard group (*n* = 11)**	***P*-value**	**Confidence interval for the difference**
Adherence to fluid intake, % of study days; median [25–75th percentiles]	75.0 [50.0–96.4]	66.7 [40.0–100.0]	0.474	−19 to 35; median difference: 4.5
Calorie intake, kcal/kg; median [25–75th percentiles]	67.9 [51.5-74.0]	67.2 [58.0-75.2]	0.878	−17.3 to 10.7; median difference: −0.7
Protein intake, gr protein/kg; median [25–75th percentiles]	1.6 [1.3–1.8]	1.5 [1.2–1.7]	0.598	−0.2 to 0.4; median difference: 0.1
Cumulative fluid intake day 3, ml/kg; mean ± SD	262.2 (±58.9)	360.5 (±52.6)	<0.001	−146.8 to −49.7; effect size: 1.8
Cumulative furosemide day 3, mg/kg; median [25^−^75th percentiles]	0.9 [0.08–2.2]	0.5 [0.0–1.0]	0.361	−0.5 to 1.5; median difference: 0.3
Cumulative diuresis at day 3, in ml/kg; mean ±SD	174.1 (±55.5)	265.0 (±36.6)	<0.001	−132.1 to −49.8; effect size: 1.9
Patients who received fluid boluses, N (%)	3 (25)	3 (26)	0.901	OR 0.89; 0.14–5.73
Acute Kidney Injury (AKI), grade I, *N* (%)	4 (33)	0 (0)	0.093	n.a.
Difference mean BP day 3–day 1, in mmHg, mean ±SD	−6.2 (±10.9)	1.9 (±10.4)	0.098	−1.6 to 17.9; effect size: 0.8
Difference heart rate day 3–day 1, in beats/min, mean ±SD	−13.0 (±22.2)	3.2 (±19.9)	0.081	−2.2 to −34.5; effect size: 0.8

*No significant differences in the feasibility and safety parameters occurred between the two groups. Diuresis was significantly higher in the standard group. Parametric data was analyzed using an independent-samples t-test. Non-parametric data was analyzed using a Mann-Whitney U test. Proportions were analyzed using logistic regression, or if count was zero: Fisher's exact test*.

**Figure 2:**
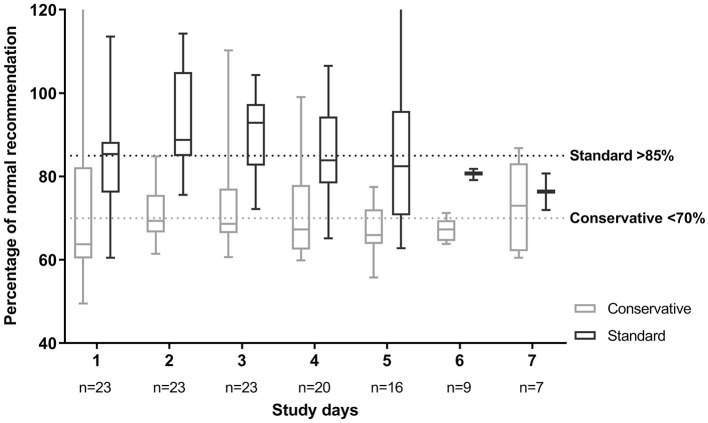
Fluid intake per fluid strategy in percentage of normal fluid recommendation. Normal fluid recommendations are based on Shaw (19). Patients in the standard fluid strategy were allocated to stay above 85% of this fluid volume. Patients in the conservative fluid strategy were deemed to stay below 70% of this recommendation. Plot depicts median with 25 and 75th percentiles and the whiskers represent minimum and maximum, over all patients in each group. This shows the large spread per day between patients.

Figure 2 portrays adherence in percentage of the normal fluid intake recommendations, which were also used for developing the fluid arms (Supplementary Table 1). Patients in the conservative arm needed to stay below 70% of this recommendation, while patients in the standard fluid strategy were deemed to receive fluids above 85% of this recommendation. Of the patients in the conservative fluid strategy group who ran over their target intake, in 78% of these instances, it was due to too high parenteral intake either because of high medication needs (43%), receiving extra glucose parenterally when enteral feeding was not yet well-tolerated (29%) or occasionally extra fluid boluses (21%) were given.

Calorie and protein intake were not significantly different between fluid strategy groups and reached a median of 67.9 [25–75th percentiles; 51.5–74.0] kcal/kg and 1.6 [1.3–1.8] gr protein/kg in the conservative and 67.2 [25–75th percentiles; 58.0–75.2] kcal/kg and 1.5 [1.2–1.7] gr protein/kg in the standard group (*p* = 0.878 for calorie intake, *p* = 0.598 for protein intake).

**Figure 3:**
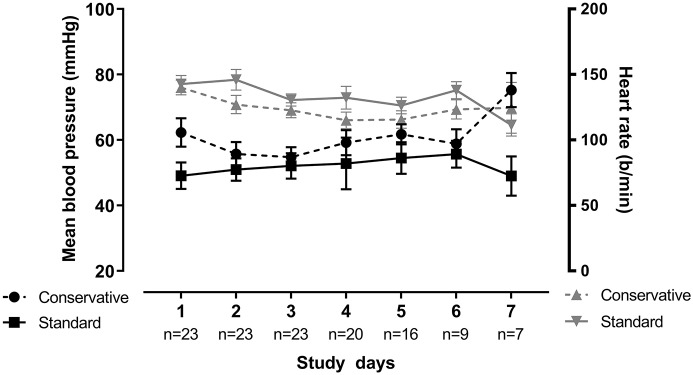
Blood pressure and heart rate over time. Blood pressure and heart rate for both groups portrayed over time. Blood pressure is found on the left Y-axis, heart rate on the right Y-axis. Plot depicts mean with the whiskers representing liberal error of the mean, over all patients in each group. There was no significant difference between groups over time for both parameters as tested by linear-mixed model (*p* = 0.687 for BP, *p* = 0.387 for HR).

The need for interventions such as administration of diuretics or fluid boluses was similar in both fluid groups. Yet cumulative diuresis was significantly higher in the standard fluid group (Cohen's *d* effect size: 1.9, 96.1% confidence interval −132.1 to −49.8; *p* < 0.001). Importantly, a conservative fluid strategy did not lead to worsened hemodynamic response over time, as measured by mean blood pressure, during the mechanical ventilation period (linear mixed model; *p* = 0.687). Change in blood pressure and heart rate was also assessed by the delta (change from day 1 to day 3) which similarly showed no significant difference (Table 2 and Figure 3). There were no significant differences in electrolyte imbalances between the fluid strategies (Table 3).

**Table 3:** Electrolyte imbalances.

**Parameter**	**Conservative group (*n* = 12)**	**Standard group (*n* = 11)**	***P*-value**
Hyponatremia, *N* (%)	5 (42)	3 (27)	0.667
Hypernatremia, *N* (%)	1 (8)	0 (0)	1.000
Hypokalemia, *N* (%)	2 (17)	0 (0)	0.478
Hyperkalemia, *N* (%)	3 (25)	4 (36)	0.667
Hypochloremia, *N* (%)	4 (33)	1 (9)	0.317
Hyperchloremia, *N* (%)	0 (0)	0 (0)	-

*No significant differences in the occurrence of imbalances were found between the two groups. Electrolytes were deemed out of balance when values were out of the normal range for more than one day consecutively. Normal ranges used were: sodium 135–145 mmol/L; potassium 3.5–5.2 mmol/L; chloride 96–111 mmol/L. Differences in occurrence of electrolyte imbalances were tested using the Fisher's exact test*.

### Fluid Intake

**Figure 4:**
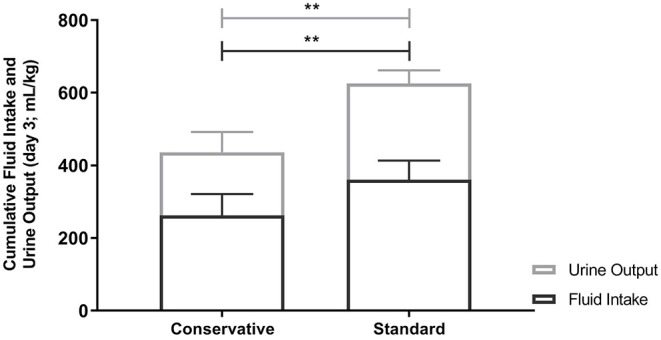
Cumulative Fluid intake and Urine output on Day 3. Fluid intake and urine output were significantly different between the conservative and standard fluid arm (*p* < 0.001). This did however not result in a difference in fluid balance as can be deducted from this figure. Bar graphs depict mean and SD. Differences between groups were tested using independent-samples *t*-tests. ^**^ *p*<0.001.

There was a significant difference in fluid intake between the allocated fluid strategies with a mean (±SD) of 262.2 (±58.9) ml/kg in the conservative group, vs. 360.5 (±52.6) ml/kg in the standard fluid group (*p* < 0.001, effect size Cohen's *d* = 1.8, Table 2). Despite this, there was no significant difference in CFB3 [mean (±SD) 79.7 (±19.7) vs. mean 84.2 (±33.0) ml/kg; effect size Cohen's *d* = 0.17, 95% confidence interval for the difference = −28.0 to 18.7, *p* = 0.682 by independent-samples *t*-test, Figure 4]. Body weight on day 3 of mechanical ventilation was available for 11 children (48% of all study patients) and for these patients the change in body weight over the first 3 days of admission could be calculated. Measuring body weight is considered quite burdensome for mechanically ventilated critically ill patients, which led to the low percentage of available weights at day 3. In the conservative group, the median weight change was +3.1% [25–75th percentiles −0.6 to 8.1] vs. +1.8% [25–75th percentiles −1.6–10.3] in the standard group (Mann-Whitney *U*-test; *p* = 1.000). Cumulative parenteral fluid intake on day 3 consisted of mean (±SD) 88.8 (±29.6) ml/kg vs. mean 115.6 (±39.7) ml/kg in the conservative and standard fluid strategy, respectively (independent-samples *t*-test; *p* = 0.079), translating into a percentage of 34 and 32% of total CFI on day 3 in either group. Urine output was significantly higher in the standard group (independent-samples *t*-test; effect size Cohen's *d* = 1.9; *p* < 0.001, Table 3 and Figure 4). In the conservative group, 4 out of 12 patients (33%) had a diuresis of <0.5 ml/kg/h for 8 h consecutively, defining AKI stage I (22), opposed to zero patients in the standard group (Fisher's exact test; *p* = 0.093; Table 2).

### Clinical Outcome

The duration of mechanical ventilation was 5.1 [25–75th percentiles 4.7–7.0] days in the conservative group and 4.3 [25–75th percentiles 2.7–5.4] days in the standard fluid group (Mann-Whitney *U* test; confidence interval for the difference = 0.04–2.87; *p* = 0.044). There was no significant difference between fluid strategies in oxygenation failure over time, as measured by OSI (linear mixed model; *p* = 0.617).

## Discussion

In this single center feasibility study we investigated the feasibility of conducting a large-scale trial comparing the current standard, more liberal, fluid maintenance strategy to a conservative fluid strategy during mechanical ventilation in critically ill pediatric patients with ARTI. Employing a protocol based on real-life practice, without extensive algorithms or flowcharts, we were able to significantly reduce fluid intake in these critically ill mechanically ventilated children, wherein moderate adherence was reached, without evidence of impairment of calorie and protein intake. In current literature, the association of fluid overload with clinical outcome in critically ill children has only been estimated through retrospective cohort studies (2, 6, 26, 27). This study provides a first instigation in research toward determining a possible causative relationship between fluid overload and worse outcome in the PICU.

Focusing on feasibility, we found that a vast majority of patients reached their targeted fluid intake. When fluid intake exceeded the targeted intake, this was mostly due to too high parenteral load if patients did not yet tolerate the necessary enteral feeding or if they had high medication needs. Although care was taken to minimize volume and concentrate all given solutions, it remains possible that errors were made and further restriction is possible in the future. Important pillars in determining feasibility are numerous safety parameters, such as nutritional intake. It seems that feeding was adequately concentrated in the conservative patients, resulting in a similar calorie and protein intake in both groups.

Different fluid strategies led to a large difference in fluid intake, yet surprisingly no difference in CFB or weight gain was reached. Although patients in the standard arm did not receive higher dosages of diuretics, they did have a significantly higher cumulative diuresis. This indicates that patients were still capable of losing excess fluids without the aid of additional medication. It would have been interesting to assess blood urea nitrogen/ creatinine ratios for these patients to assess kidney function in more depth. However, this was not available for most patients, as it was not protocolized to be determined standardly in our PICU. The similar fluid balances in both fluid groups, suggest that patients in the standard arm received more fluid than was necessary for their clinical situation. In this cohort more than 70% of the patients were infants with respiratory failure due to viral-induced ARTI, which is in general a population without multiple organ failure (including severe kidney injury) and/or major capillary leak. Apparently, these patients are still able to maintain adequate diuresis and fluid homeostasis. Yet, in an earlier study we found that this specific patient group also suffers from fluid overload which is associated with prolonged duration of mechanical ventilation (8). Nevertheless, this study was not powered to assess the effect of fluid strategy on clinical outcome and further research is required to draw further conclusions on this matter. Taken together, these outcomes suggest that there might be room for further fluid restriction, possibly by combining low fluid volume administration with early start of active diuretic treatment. Although we cannot draw conclusions of the effect of these specific fluid strategies for other patient populations based on this study, it could be hypothesized that patients with sepsis or severe acute respiratory distress syndrome and therefore more risk of capillary leak and/or AKI, will show a different response in terms of fluid balance upon use of similar fluid strategies. A recent implementation study in children with acute respiratory distress syndrome or sepsis has shown that the combination of fluid restriction, drug volume reduction, dynamic monitoring of preload markers to determine the need for fluid bolus administration, early use of diuretics and early initiation of enteral feeds decreased fluid overload and mechanical ventilation duration (28). However, this study was a retrospective cohort and a future RCT in the future might provide more robust evidence. As the authors themselves pointed out selection bias and type-II error cannot be ruled out. In our study we found a small significant difference in mechanical ventilation duration favoring the standard fluid treatment over conservative fluids. Yet, the confidence intervals of this difference were quite wide, with a lower limit close to 0, indicating that more research is required to narrow the confidence intervals. Importantly, in the intervention group (conservative fluids), there were 4 patients with a history of chronic illness, possibly contributing to the longer duration of mechanical ventilation in this group. If indeed, a conservative fluid regimen has a negative impact on mechanical ventilation the benefits and harms of a conservative fluid strategy should be carefully considered.

In order to set up a multicenter RCT in the future, we need to take a close look at the needed sample size to obtain a sufficiently precise answer whether a conservative fluid strategy could provide clinical benefit in this pediatric population. We consider 1 day a minimum clinically important difference (MCID) in duration of ventilation. For this study we obtained a common SD of the parameter of ventilation duration of 1.8. With a significance level of 0.05 and power of 80%, this would require 52 patients per group. In an earlier retrospective study of mechanically ventilated patients with ARTI, we found a SD of 3.0. When taking this SD into account, with similar MCID, a sample size of 143 patients per group would be needed. Of course we have to take into account that if a future RCT were to include a more heterogeneous group of patients, an even wider spread in mechanical ventilation duration is expected and the sample size needed may increase even further. Another side note which is essential to consider, is that in this study we did not yet succeed in influencing the CFB3, which will be the primary goal for any future RCT since the CFB3 is the marker associated with adverse outcome in earlier literature. Although we might be able to accomplish this with further fluid restriction, we were not able to take this into account while calculating abovementioned sample size.

We believe that the main strength of our study is that it is pragmatically designed study, using a standardized protocol that was based on current clinical practice in critically ill children, that are prone to various risks of fluid overload. Due to this design, implementation into the clinical practice of potential study results is both relevant and feasible. Second, we were able to select a homogeneous study population with a circumscript disease entity that is frequently seen in PICU settings. Finally, we were able to prove feasibility of a larger RCT on this topic. A limitation of our study is the small sample size, Yet, according to the published literature on minimal sample size for pilot studies we did obtain sufficient patients per group (17, 18). Nevertheless, given our sample size and following the rule of three, we would have at least a 95% chance to detect at least one adverse event for an occurrence rate of adverse events of 13% (1 in 8 patients) or higher. This is of course limited, so a future RCT will have to confirm the safety, along with the effect on fluid overload and clinical outcome. Another limitation is that there is quite a wide variance in adherence to fluid allocation, which is an important point of focus for a future confirmatory RCT.

Elaborating on the data from this study, the next question is how to set up the different fluid strategies for a future multicenter RCT. This study suggests that a conservative fluid management strategy is feasible and safe both hemodynamically and with regard to nutritional intake. Moreover, the standard, liberal fluid strategy led to higher diuresis but similar fluid balances, making it a potential harmful form of overtreating our patients. This study implies that we really need to work on further fluid restriction (i.e., using diuretics) to decrease the CFB, as that seems to be our primary goal given the association with adverse outcome in literature. We propose a conservative fluid regimen which consists of compensation for insensible losses, which are significantly lower in mechanically ventilated patients (29), and a surplus of volume for medication, feeding, and securing kidney function. Securing kidney function should be treated with caution to prevent (severe) AKI from occurring. In addition to fluid volume restriction, early start of active diuretic treatment should be considered. Altogether, this creates a tailor-made and patient-targeted fluid management strategy. How to optimize enrolment and adherence is one of the other factors to consider when setting up the larger RCT. We experienced a decline rate of about 18%, which stresses the need for optimal counseling of parents. Moreover, specific attention should be given to maximize the adherence to the protocol. Extended instruction sessions and provision of study information for the collaborating hospitals are crucial in this. Lastly, careful consideration should be given to the inclusion of specific patient populations. This feasibility study included only patients with ARTI, which usually present with a relatively mild disease with low occurrence of multiple organ failure. Patients who are more severely affected, such as patients with severe acute respiratory distress syndrome or sepsis, might benefit even more from a conservative fluid regimen as they are more prone to capillary leak and the development of fluid overload. Therefore, in any future trial, it should be considered to include a wider population of PICU patients in order to more adequately study the effect of fluid regimen on outcome.

In conclusion, a conservative fluid strategy in mechanically ventilated children with ARTI seems feasible, without limiting metabolic needs. Future recommendations would be to conduct a large multicenter RCT in this specific patient population, be it with an adapted protocol of further fluid restriction and early start of active diuresis treatment.

## Data Availability Statement

The datasets generated for this study are available on request to the corresponding author.

## Ethics Statement

This study was approved by the Medical Ethical Review board of the Amsterdam UMC [NL55053.018.16]. Written informed consent for participation was obtained from parents or guardians of eligible patients.

## Author Contributions

SI designed the study, included and randomized patients, and optimized their fluid intake accordingly, analyzed and interpreted the data and wrote the manuscript. VG helped design the study specifically focusing on the calorie and protein intake requirements. Moreover, he contributed in writing the manuscript. MD focused specifically on designing the calorie and protein intake requirements and provided advice during the study on how to optimize energy intake during study participation. JL interpreted the data and provided major contribution in writing the manuscript. RB designed the study, interpreted the data and was a major contributor in writing the manuscript. JW designed the study, interpreted the data and also provided major contribution in writing the manuscript. All authors read and approved the final manuscript.

### Conflict of Interest

The authors declare that the research was conducted in the absence of any commercial or financial relationships that could be construed as a potential conflict of interest.
