# Supplementary material for: Less Is More?—A Feasibility Study of Fluid Strategy in Critically Ill Children With Acute Respiratory Tract Infection
Source: Front Pediatr. 2019 Dec 10;7:496. doi: 10.3389/fped.2019.00496 (PMC6915071; doi:10.3389/fped.2019.00496)
Supplement: Supplementary file 1 [file Table_1.DOCX]

**Online Supplement: Table S1**

| **Weight (kg)** |  | **Normal fluid recommendations (ml/kg/day)** | **Conservative: 70% fluid restriction**  **(ml/kg/day)** | **Standard: >85% of normal recommendations  (ml/kg/day)** | **Guarantee of kcal intake (kcal/kg/day)** | **Guarantee of protein intake (g/kg/day)** |
| --- | --- | --- | --- | --- | --- | --- |
| 0 - 6 |  | 150 | 105 | >128 | 75 | 1.5 |
| 6 - 7.5 |  | 130 | 91 | >111 | 75 | 1.5 |
| 7.5 - 9 |  | 120 | 84 | >102 | 75 | 1.5 |
| 9 - 10 |  | 110 | 77 | >94 | 75 | 1.5 |
| 11 |  | 95 | 67 | >81 | 60 | 1.5 |
| 12 |  | 92 | 64 | >78 | 60 | 1.5 |
| 13 |  | 88 | 62 | >75 | 60 | 1.5 |
| 14 |  | 86 | 60 | >73 | 60 | 1.5 |
| 15 |  | 83 | 58 | >71 | 60 | 1.5 |
| 16 |  | 81 | 57 | >69 | 60 | 1.5 |
| 17 |  | 79 | 56 | >67 | 60 | 1.5 |
| 18 |  | 78 | 54 | >66 | 60 | 1.5 |
| 19 |  | 76 | 53 | >65 | 60 | 1.5 |
| 20 |  | 75 | 53 | >64 | 60 | 1.5 |
| 21 |  | 72 | 51 | >61 | 60 | 1.5 |
| 22 |  | 70 | 49 | >60 | 60 | 1.5 |
| 23 |  | 68 | 47 | >58 | 60 | 1.5 |
| 24 |  | 66 | 46 | >56 | 60 | 1.5 |
| 25 |  | 64 | 45 | >54 | 60 | 1.5 |
| 26 |  | 62 | 44 | >53 | 60 | 1.5 |
| 27 |  | 61 | 43 | >52 | 60 | 1.5 |
| 28 |  | 59 | 42 | >50 | 60 | 1.5 |
| 29 |  | 58 | 41 | >49 | 60 | 1.5 |
| 30 |  | 57 | 40 | >48 | 60 | 1.5 |
| >30 |  | 50 | 35 | >43 | 60 | 1.5 |

**Supplementary Table S1: Fluid strategies per randomization arm per kg bodyweight**

Normal fluid recommendations of infants and young children are based on Shaw (19).
